# Retrospective analysis of infliximab and adalimumab treatment in a large cohort of juvenile dermatomyositis patients

**DOI:** 10.1186/s13075-020-02164-5

**Published:** 2020-04-15

**Authors:** Raquel Campanilho-Marques, Claire T. Deakin, Stefania Simou, Charalampia Papadopoulou, Lucy R. Wedderburn, Clarissa A. Pilkington, for the Juvenile Dermatomyositis Research Group (JDRG), Kate Armon, Joe Ellis-Gage, Holly Roper, Vanja Briggs, Joanna Watts, Liza McCann, Ian Roberts, Eileen Baildam, Louise Hanna, Olivia Lloyd, Susan Wadeson, Michelle Andrews, Phil Riley, Ann McGovern, Verna Cuthbert, Clive Ryder, Janis Scott, Beverley Thomas, Taunton Southwood, Eslam Al-Abadi, Ruth Howman, Sue Wyatt, Gillian Jackson, Mark Wood, Tania Amin, Vanessa VanRooyen, Deborah Burton, Louise Turner, Sarah Hanson, Joyce Davidson, Janet Gardner-Medwin, Neil Martin, Sue Ferguson, Liz Waxman, Michael Browne, Roisin Boyle, Emily Blyth, Mark Friswell, Helen Foster, Alison Swift, Sharmila Jandial, Vicky Stevenson, Debbie Wade, Ethan Sen, Eve Smith, Lisa Qiao, Stuart Watson, Claire Duong, Helen Venning, Rangaraj Satyapal, Elizabeth Stretton, Mary Jordan, Ellen Mosley, Anna Frost, Lindsay Crate, Kishore Warrier, Stefanie Stafford, Kelly Sandhu, Tracey Dandy, Lucy Wedderburn, Clarissa Pilkington, Nathan Hasson, Muthana Al-Obadi, Giulia Varnier, Sandrine Lacassagne, Sue Maillard, Lauren Stone, Elizabeth Halkon, Virginia Brown, Audrey Juggins, Sally Smith, Sian Lunt, Elli Enayat, Hemlata Varsani, Laura Kassoumeri, Laura Beard, Katie Arnold, Yvonne Glackin, Stephanie Simou, Beverley Almeida, Kiran Nistala, Raquel Marques, Claire Deakin, Parichat Khaosut, Stefanie Dowle, Charalampia Papadopoulou, Shireena Yasin, Christina Boros, Meredyth Wilkinson, Chris Piper, Cerise Johnson-Moore, Lucy Marshall, Kathryn O’Brien, Emily Robinson, Kevin Murray, Coziana Ciurtin, John Ioannou, Caitlin Clifford, Linda Suffield, Helen Lee, Sam Leach, Helen Smith, Anne-Marie McMahon, Heather Chisem, Jeanette Hall, Ruth Kingshott, Maxine Mutten, Nick Wilkinson, Emma Inness, Eunice Kendall, David Mayers, Ruth Etherton, Danielle Miller, Kathryn Bailey, Jacqui Clinch, Natalie Fineman, Helen Pluess-Hall, Suzanne Sketchley, Joyce Davidson, Margaret Connon, Lindsay Vallance, Kirsty Haslam, Charlene Bass-Woodcock, Trudy Booth, Louise Akeroyd, Alice Leahy, Amy Collier, Rebecca Cutts, Emma Macleod, Hans De Graaf, Brian Davidson, Sarah Hartfree, Danny Pratt, Elizabeth Fofana, Lorena Caruana

**Affiliations:** 1 Infection, Inflammation and Rheumatology Section, UCL Great Ormond Street Institute for Child Health, London, UK; 2 Rheumatology Section, Great Ormond Street Hospital for Children NHS Trust, Level 6 Southwood Building, Great Ormond Street, London, WC1N 3JH UK; 3 NIHR Biomedical Research Centre at Great Ormond Street Hospital for Children NHS Foundation Trust, London, UK; 4 Serviço de Reumatologia e Doenças Ósseas Metabólicas, Hospital de Santa Maria, CHULN—Centro Académico de Medicina de Lisboa, Lisbon, Portugal; 5 Unidade de Investigação em Reumatologia, Instituto de Medicina Molecular, Faculdade de Medicina, Universidade de Lisboa—Centro Académico de Medicina de Lisboa, Lisbon, Portugal; 6 Centre for Adolescent Rheumatology Versus Arthritis at UCL, UCLH and GOSH, London, UK

**Keywords:** Infliximab, Adalimumab, Juvenile dermatomyositis, Biologic therapy, P rheumatology

## Abstract

**Background:**

Anti-TNF treatment may be useful for the treatment of patients with refractory juvenile dermatomyositis (JDM). The aim of this study was to describe the use of infliximab and adalimumab therapy in juvenile dermatomyositis as an adjunctive treatment.

**Methods:**

Sixty children recruited to the UK JDM Cohort and Biomarker Study that had received at least 3 months of anti-TNF treatment (infliximab or adalimumab) were studied. Childhood Myositis Assessment Scale (CMAS), Manual Muscle Testing (MMT8) and physician’s global assessment (PGA) were recorded. Skin disease was assessed using the modified skin disease activity score (DAS). Data were analysed using Friedman’s test for repeated measures analysis of variance.

**Results:**

Compared to baseline, there were improvements at 6 and 12 months in skin disease (*χ*^2^(2) = 15.52, *p* = 0.00043), global disease (*χ*^2^(2) = 8.14, *p* = 0.017) and muscle disease (CMAS *χ*^2^(2) = 17.02, *p* = 0.0002 and MMT *χ*^2^(2) = 10.56, *p* = 0.005) in infliximab patients. For patients who switched from infliximab to adalimumab, there was improvement in global disease activity (*χ*^2^(2) = 6.73, *p* = 0.03), and trends towards improvement in CMAS, MMT8 and modified DAS. The median initial prednisolone dose was 6 [0–10] mg, and final was 2.5 [0–7.5] mg (*p* < 0.0001). Fifty-four per cent of patients had a reduction in the number and/or size of calcinosis lesions. Twenty-five per cent switched their anti-TNF treatment from infliximab to adalimumab. 66.7%of the switches were to improve disease control, 26.7% due to adverse events and 6.6% due to patient preference. A total of 13.9 adverse reactions occurred in 100 patient-years, of which 5.7 were considered serious.

**Conclusion:**

Reductions in muscle and skin disease, including calcinosis, were seen following treatment with infliximab and adalimumab.

## Background

Juvenile dermatomyositis (JDM) is a rare inflammatory disease of childhood that predominantly affects muscles and skin but is also a systemic multi-organ disease [1]. It is the most common idiopathic inflammatory myopathy (IIM) of childhood: incidence of 2–3 new cases per million/children/year [2]. The treatment of JDM has been challenging. The reasons include the rarity of the disease, its heterogeneous clinical phenotype and the small number of randomized, double-blind controlled clinical trials. Traditional treatment includes glucocorticoids and conventional immunosuppressive or immunomodulatory agents [3]. As treatment of refractory disease has been difficult, there is growing interest in evaluating novel therapies including newer biologics that target various pathways implicated in the pathogenesis of myositis [4]..

Anti-TNF biologics have been successful in treating various chronic inflammatory disorders [5, 6]. TNF has been identified in high levels in JDM patients who have a long disease course and calcinosis, which can be a debilitating complication [7–9]. There is some evidence that prolonged active disease is related to this complication and that its incidence can be reduced by earlier disease control [10]. A previous case series of 5 patients with refractory JDM treated with infliximab showed improvement in all 5 cases in core set measures [11]. Therefore, TNF may be a good potential therapeutic target for the treatment of JDM: however, evidence for efficacy of TNF blockade is limited. The goal of this study was to describe the efficacy and safety of anti-TNF treatment in patients recruited to the UK JDM Cohort and Biomarker Study (JDCBS).

## Methods

### Patients

Data from JDM patients treated with anti-TNF agents were analysed from the JDCBS [2]. Informed written parental consent and age-appropriate assent were obtained. This research was approved by the UK Northern & Yorkshire Medical Research and Ethics Committee. All patients had a diagnosis of definite or probable JDM according to the Bohan and Peter criteria [12, 13]. The new classification criteria were not employed as they are not yet used in routine clinical practice. Patients were included in the analysis if they had ever received anti-TNF therapy lasting for at least 3 months. Patients were excluded from the analysis if they were treated with etanercept or had no recorded outcome variables or dates of starting or ending anti-TNF. Indications for starting anti-TNF were active skin disease, calcinosis, muscle disease or general disease activity that the clinician considered to be refractory to conventional treatment.

Sixty patients treated with anti-TNF were identified, and data on the demographics, concomitant disease-modifying anti-rheumatic drugs (DMARDs) and adverse events are presented for this combined cohort in order to analyse treatments targeting this mechanism as a whole. Patients were excluded from subsequent efficacy analyses if they had allergic reactions to the first infusion and did not receive another anti-TNF (*n* = 2). For efficacy analysis, patients treated with infliximab alone (6 mg/kg every 4 weeks; *n* = 39) and patients treated with infliximab (6 mg/kg every 4 weeks) then adalimumab (24 mg/m^2^ every other week; *n* = 15) were analysed as two separate groups. Changes in levels of disease activity at 6 and 12 months after infliximab and adalimumab start were analysed for the respective groups. Finally, patients treated with adalimumab alone (24 mg/m^2^ every other week; *n* = 4) were not analysed statistically due to low numbers, but their clinical scores are described. A flow diagram outlining which patients were included in each analysis is presented Figure S1 in Supplementary Methods. Although patients who received etanercept were excluded from analysis, a description of their clinical scores is presented in Supplementary Results.

### Data collection

Patient clinical data at 0, 6 and 12 months after infliximab/adalimumab start are described. For the safety analysis, patient clinical data were collected until the present date. Core outcome variables for JDM were collected including the Childhood Myositis Assessment Scale (CMAS, score range 0–53, with high scores indicating minimal disease) [14] and Manual Muscle Testing of 8 groups (MMT8, score range 0–80, with high scores indicating minimal disease) [15] that exist to standardize the muscle assessment. Both are part of the JDM Paediatric Rheumatology International Trials Organisation (PRINTO) [16] and International Myositis Assessment and Clinical Studies Group (IMACS) [17] core disease activity measures. The physician’s global assessment of disease activity (PGA, score range 0–10, with low scores indicating minimal disease) and the modified Disease Activity Score as a measure of skin disease activity (DAS, score range 0–5, with low scores indicating minimal disease) [18] were collected. Modified DAS includes the 4 skin components of a simplified version of the original DAS tool, which are Gottron’s papules (1 point), heliotrope rash (1 point), vasculitis (1 point) and erythema (2 points). The presence of calcinosis was also recorded based on a combination of physician’s assessment and imaging such as X-rays. As objective measures of calcinosis were not collected for this cohort, analyses of whether calcinosis improved were based on the physician’s assessment at the date of visit, which may have incorporated X-rays. Inter-observer variation was not recorded. Data on other medications received prior to the start, at start and at 12 months of anti-TNF therapy were recorded, including the dose of steroid treatment. Adverse events that occurred from the start of anti-TNF treatment until the end of follow-up were recorded. Severe adverse reactions were defined as the occurrence of death, hospitalization or any event that caused permanent damage. For patients who switched between anti-TNF therapies (infliximab to adalimumab), the reason for the switch and the duration of prior infliximab treatment at the time of the switch were noted.

### Statistical analysis

The non-parametric Friedman’s test for repeated measures analysis of variance (ANOVA) was performed to identify significant main effects of time on clinical measures of disease activity. Separate analyses were performed for patients who received infliximab alone and for patients who received infliximab and then adalimumab. In the latter analysis, the date of switch from infliximab to adalimumab was taken as a new treatment start time. Post hoc tests to identify time-points at which clinical scores differed significantly from each other were performed using Wilcoxon signed-rank tests. The Bonferroni correction was used to adjust for multiple hypothesis testing, such that *p* values below 0.017 were considered statistically significant in the post hoc tests. Steroid doses at the start and 12 months after the start of anti-TNF therapy were compared using the Wilcoxon signed-rank test. Statistical analysis was performed using R version 3.5.1 and plots were generated using Graphpad Prism 5.

## Results

### Patient demographics, clinical features and medication

Of 60 patients assessed, 72% were female and 77% were Caucasian (Table 1). Most had been diagnosed with definite JDM (87%), with a minority diagnosed with probable JDM (3%), JDM overlap with scleroderma (7%) or JDM overlap with chronic arthritis (2%) The median age at disease onset was 5.2 [3.3–9.7] years. Median disease duration at the beginning of anti-TNF treatment was 3.1 [1.7–4.9] years, and median duration on anti-TNF therapy was of 2.5 [1.5–4] years. Of these patients, 59 had an autoantibody result: 19 (32%) had anti-TIF1γ, 7 (12%) had anti-NXP2, 1 (2%) had anti-MDA5, 1 (2%) had anti-Mi2, 1 (2%) had anti-SRP, 1 (2%) had anti-PL-7 and 1 (2%) had anti-HMGCR myositis-specific autoantibodies. A further 2 (3%) had anti-PMScl, and 1 (2%) had anti-Topo myositis-associated autoantibodies. One patient (2%) had both anti-U1RNP and anti-TIF1γ autoantibodies, 13 (22%) had unidentified autoantibodies and 11 (19%) had no-detectable autoantibodies.

**Table 1:** Demographic and serological features of patients who received anti-TNF therapy (*n* = 60)

Feature	Number (%) or median [IQR]
Sex	
Male	17 (28%)
Female	43 (72%)
Ethnicity	
Caucasian	46 (77%)
Non-Caucasian	14 (23%)
Diagnosis	
Definite JDM	52 (87%)
Probable JDM	2 (3%)
JDM overlap with scleroderma	4 (7%)
JDM overlap with chronic arthritis	1 (2%)
Age at disease onset	5.2 [3.3–9.7]
Disease duration at anti-TNF start	3.1 [1.7–4.9]
Duration on anti-TNF therapy	2.5 [1.5–4]
Autoantibody	
Anti-TIF1γ	19 (32%)
Anti-NXP2	7 (12%)
Anti-MDA5	1 (2%)
Anti-Mi2	1 (2%)
Anti-SRP	1 (2%)
Anti-PL-7	1 (2%)
Anti-HMGCR	1 (2%)
Anti-PMScl	2 (3%)
Anti-Topo	1 (2%)
Anti-U1RNP and anti-TIF1γ	1 (2%)
Unidentified autoantibodies	13 (22%)
No-detectable autoantibodies	11 (19%)

Regarding prior treatment, 98% of the patients were on methotrexate, azathioprine or hydroxychloroquine in monotherapy or in combination before starting anti-TNF (Table 2). Steroids were given prior to anti-TNF treatment in 92% of patients; of those with available data, 68% were still on steroids at anti-TNF start consistent with recalcitrant disease. Intravenous immunoglobulin (IVIg) treatment had been received by 11% of the patients prior to anti-TNF treatment, whilst 13% were still on IVIg at the start of the anti-TNF treatment.

**Table 2:** Treatment at time of the first assessment of the 60 patients identified

Treatment	Previously	At start of anti-TNF	After 12 months on anti-TNF
MTX/AZA/HQL^1^	48 (98%), *n* = 49	43 (80%), *n* = 56	41 (89%), *n* = 46
Cyclophosphamide	26 (43%), *n* = 60	3 (5%), *n* = 60	0, *n* = 43
Immunoglobulin	5 (11%), *n* = 47	6 (13%), *n* = 46	1 (2.5%), *n* = 40
Oral steroids	47 (92%), *n* = 51	26 (68%), *n* = 38	26 (58%), *n* = 45
2 or more DMARDs	48 (98%), *n* = 49	30 (64%), *n* = 47	

*MTX* methotrexate, *AZA* azathioprine, *HQL* hydroxychloroquine, *n* (%) absolute numbers (percentages) from the number of patients with available data

^1^Mycophenylate mofetil (MMF) was not used in the patients in this study

**Fig. 1:**
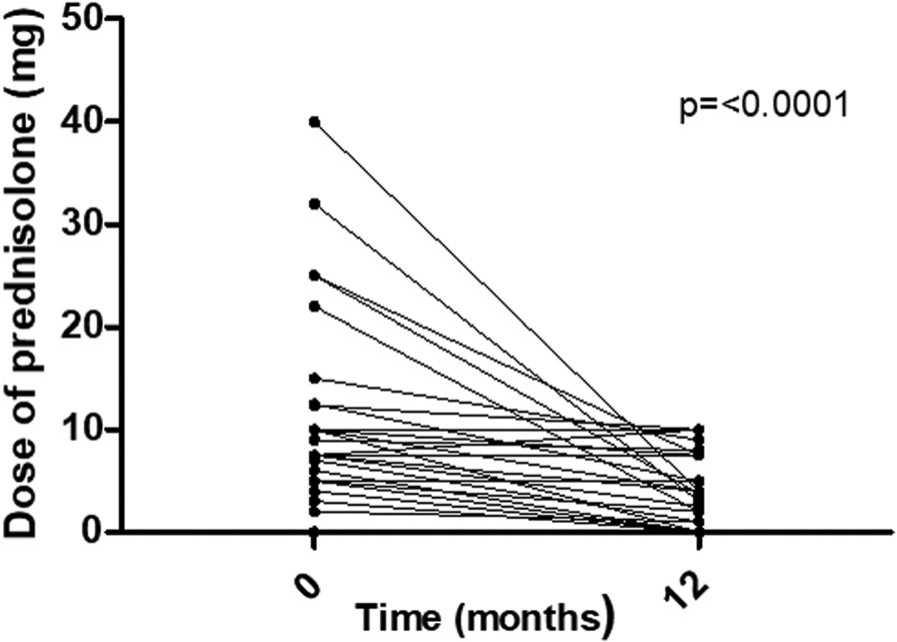
Steroid-sparing effect of use of anti-TNF therapy. Dose of prednisolone (mg/day) at anti-TNF start and 12 months of anti-TNF treatment. *n* = 43 patients (number of patients with complete data available)

43% of the patients had finished treatment with cyclophosphamide (typically 6–7 doses, administered intravenously) before receiving anti-TNF. Five per cent (*n* = 3) of the patients started anti-TNF whilst they were still on cyclophosphamide (2 patients started anti-TNF 2 to 3 weeks before finishing the last cyclophosphamide dose of the course, and only 1 patient started anti-TNF before cyclophosphamide). After 12 months of anti-TNF therapy, none were still on cyclophosphamide. The median prednisolone daily dose at anti-TNF start was 6 [0–10] mg, and after 12 months of anti-TNF treatment was 2.5 [0–7.5] mg (*p* < 0.0001; Fig. 1, complete data available on 43 patients).

### Efficacy on infliximab therapy

**Fig. 2:**
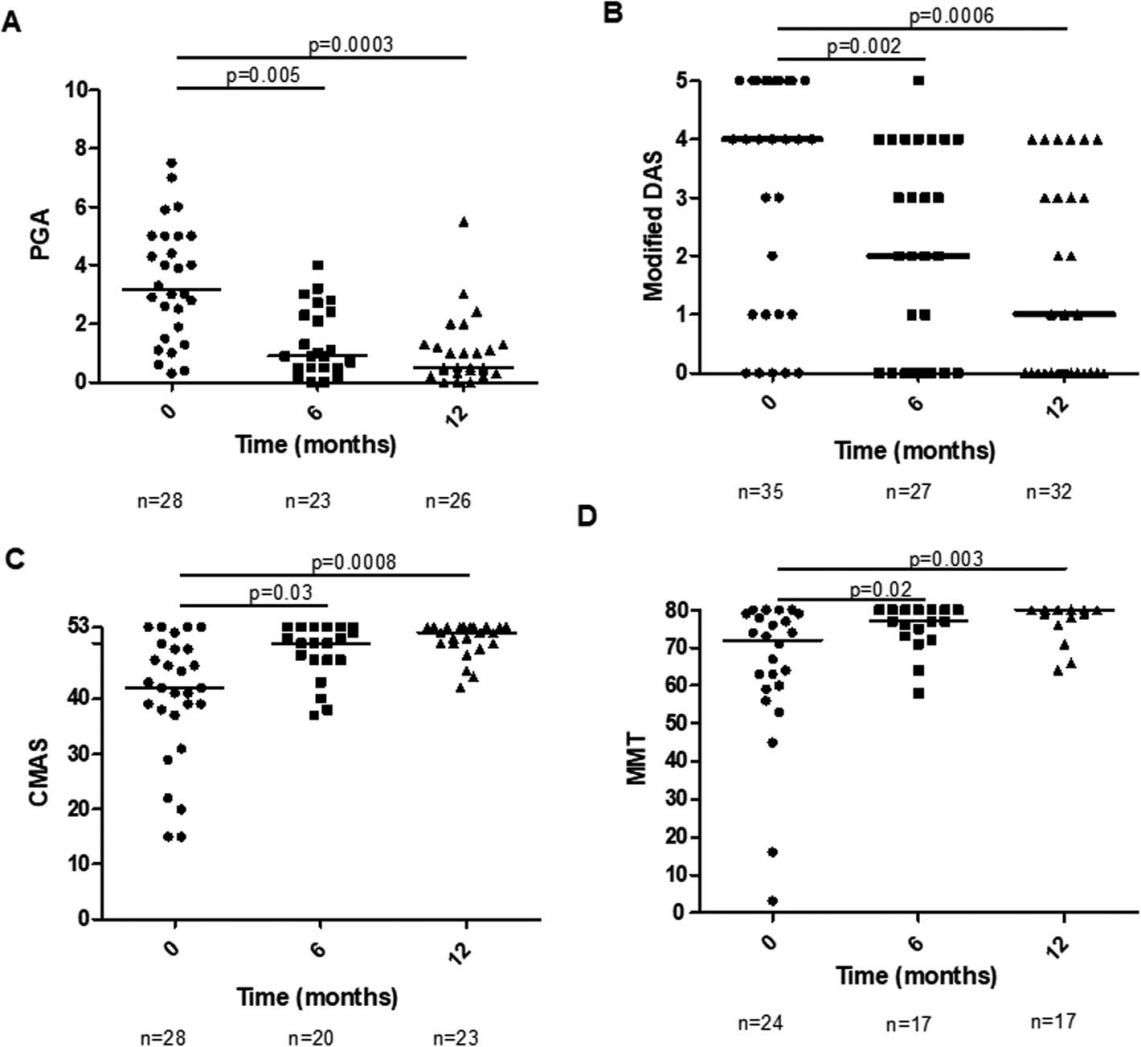
Clinical measures over time in patients treated with infliximab (total of 43 patients). Clinical outcome measures of patients on infliximab are shown at baseline (time of starting infliximab) and at 6 and 12 months of infliximab treatment. **a** PGA, **b** Modified DAS, **c** CMAS and **d** MMT. *n,* number of patients with available data; PGA, Physician Global Assessment; DAS, Disease Activity Score; CMAS, Childhood Myositis Assessment Scale; MMT, Manual Muscle Testing

In the 39 patients that received infliximab alone, global disease activity improved (*χ*^2^(2) = 8.14, *p* = 0.017; Fig. 2a). PGA decreased from 3.2 [1.8–5] at anti-TNF initiation to 0.9 [0.5–2.4] (*p* = 0.005) at 6 months and to 0.5 [0.3–1.3] at (*p* = 0.0003) 12 months. Skin involvement improved (*χ*^2^(2) = 15.52, *p* = 0.00043; Fig. 2b). Modified DAS decreased from 4 [1.5–5] at anti-TNF initiation to 2 [0–4] at 6 months (*p* = 0.002) and to 1 [0–3.3] at 12 months (*p* = 0.0006). Muscle involvement also improved in terms of CMAS and MMT 80 (*χ*^2^(2) = 17.02, *p* = 0.0002—Fig. 2c and *χ*^2^(2) = 10.56, p = 0.005—Fig. 2d, respectively). Median CMAS increased from 42 [37.8–49] at anti-TNF initiation to 50 [47–53] at 6 months (*p* = 0.03; not considered statistically significant following Bonferroni correction) and to 52 [50–53] (*p* = 0.0008) at 12 months. Median MMT 80 increased from 72 [59.8–78.3] to 77 [73–80] at 6 months (0.02; not significant) and to 80 [78–80] at 12 months (0.003).

Of the 39 patients treated with infliximab alone, 15 patients were identified who had been treated with cyclophosphamide 1.9 [0.8–2.2] years prior to starting infliximab. Indications for cyclophosphamide include severe skin disease, severe muscle weakness, severe calcinosis, widespread vasculitis and failure to respond to first-line treatment. When these patients were excluded and the remaining patients analysed (*n* = 24), improvements in disease activity were observed in the remaining patients treated with infliximab alone (*n* = 24) for skin disease activity (*χ*^2^(2) = 6.08, *p* = 0.048 for modified DAS) and muscle disease activity (*χ*^2^(2) = 10.17, *p* = 0.006 for CMAS). Modified DAS reduced from 4 [1–4.3] at infliximab start to 2 [0–3] at 6 months (*p* = 0.018, not considered significant following Bonferroni correction) and 1 [0–3] at 12 months (*p* = 0.013). CMAS increased from 44 [38.8–50.5] at anti-TNF start to 52.5 [50–53] at 6 months (*p* = 0.11) and 52 [50–53] at 12 months (*p* = 0.03, not significant).

### Efficacy after switching to adalimumab

Fifteen patients (25%) switched their anti-TNF treatment from infliximab to adalimumab. The median time of switching from infliximab to adalimumab was 2.3 months [1–3.8]. Ten (66.7%) of the switches were due to treatment inefficacy, 1 (6.6%) related to patient preference for subcutaneous administration and 4 (26.7%) were due to adverse events such as hypersensitivity reactions. From those 10 patients that switched due to treatment inefficacy, 8 were mainly due to active skin disease (5 had calcinosis lesions progressing). Only 3 of those 10 switches happened before 1 year on infliximab; all the others happened after 2 to 3 years on the drug.

**Fig. 3:**
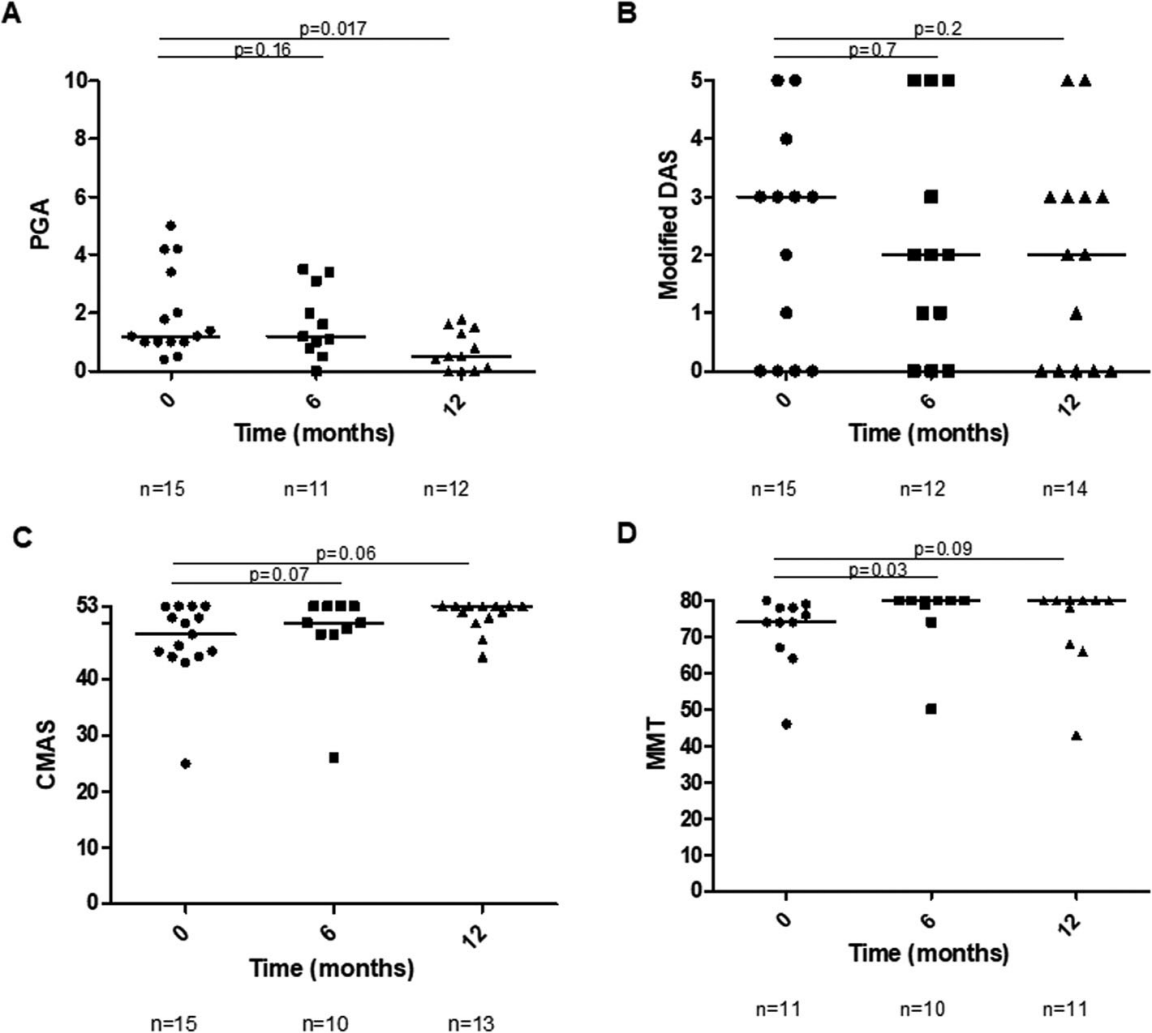
Clinical measures in patients who switched from infliximab to adalimumab (total of 16 patients). Score shown at 0 (time of switch), 6 and 12 months of Adalimumab treatment. **a** PGA, **b** Modified DAS, **c** CMAS and **d** MMT. *n*, number of patients with available data; PGA, Physician Global Assessment; DAS, Disease Activity Score; CMAS, Childhood Myositis Assessment Scale; MMT, Manual Muscle Testing

For the patients who switched from infliximab to adalimumab (*n* = 15 patients), there was improvement in global disease activity (*χ*^2^(2) = 6.73, *p* = 0.03; Fig. 3a). PGA decreased from 1.2 [1–2.7] at adalimumab initiation to 0.5 [0.1–1.4] (*p* = 0.017; borderline significant) at 12 months. There were trends towards improvement in Modified DAS, CMAS and MMT8 (Fig. 3b–d).

For the limited number of patients on adalimumab alone (*n* = 4), median physician’s VAS at anti-TNF start was 2.6 (IQR 1.8–3.4), was 1.0 (IQR 0.5–1.25) at 6 months after anti-TNF start and was 1.5 (IQR 1.38–1.8) at 12 months after anti-TNF start. Median Modified DAS at anti-TNF start was 2 (IQR 1.5–2.3), was 3 (IQR 1.5–4) at 6 months after anti-TNF start and was 1 (IQR 0–2.5) at 12 months after anti-TNF start. Median CMAS at anti-TNF start was 52 (IQR 45–52), and there was no change over the year.

### Resolution of calcinosis

Within the cohort of 60 patients, 28 patients (47%) with calcinosis were identified during their disease course up to the time of analysis. From the data recorded within the JDCBS [2] dataset, from the 28 patients, 15 (54%) had a reduction in the number and/or size of calcinotic lesions and calcinosis completely resolved in 8 (29%). Three out the 28 patients had fewer than 3 lesions which remained stable, and 3 out of the 28 patients had widespread lesions which remained stable. In 7, there were not sufficient data to evaluate changes. From the 15 patients that had a reduction in the number and/or size of calcinotic changes, we then further sub-analysed the 11 cases in which we had access to more detailed clinical information. This showed that the median time to improve was 2.75 [0.9–4] years, with a minimum of 0.25 years (3 months) and a maximum of 10 years. From those 11 patients, the calcinosis completely resolved in 4 of them in a median time of 2.8 [0.73–6.9] years and anti-TNF treatment was suspended in 3 of them with a median time of 4.6 [3–9] years after the beginning of the drug.

### Adverse events

A total of 29 adverse events were reported, of which 12 were severe adverse events: 9 were allergic reactions to infliximab (HACA levels not routinely available in the UK), and there were 3 hospital admissions of Infliximab patients (sepsis in one and pneumonia in 2). One patient died due to small bowel perforation (probably secondary to disease damage) thought to be due to calcinosis in the intestinal walls. The patient was on infliximab for 6 years and then swapped to adalimumab for nearly 1 year before time of death. The remaining adverse reactions (*n* = 17) were not severe: 14 (82.4%) were due to infectious causes (5 bacterial upper respiratory infections, 4 viral infections, 2 skin infections, 2 episodes of tonsillitis, 1 episode of chicken-pox) and 3 (17.6%%) were due to local injection site reaction (*n* = 2) and 1 episode of skin rash. In 4 of the mild to moderate adverse reactions the drug had to be discontinued (2 patients on adalimumab had injection site reactions and 2 patients on infliximab had recurrent skin infections) whilst in the remaining patients temporarily withholding the drug proved sufficient. No tuberculosis or malignancy was recorded. According to the exposure time, a total of 13.9 adverse reactions occurred by 100 patient-years. 5.7 serious adverse reactions by 100 patient-years were reported.

## Discussion

Biologic drugs have been used off-label since 2000 for JDM and other inflammatory myositis diseases with encouraging results [19]. High levels of TNF-α have been reported in JDM patients with a long disease course suggesting that it may play a significant role in refractory disease [7]. A recent study published by Spencer et al. [19], the results of a survey on CARRA members’ experience of using biologics in JDM, showed that survey responders considered that use of biologics significantly reduced complications in JDM (such as calcinosis, muscle atrophy, lipodystrophy) and were a logical therapeutic step after failure of corticosteroid and other immunosuppressive therapy, in JDM patients with resistant disease.

In our study, those who received infliximab and who switched from infliximab to adalimumab showed improvement in terms of global disease and improvement compared to their own baseline, but we cannot exclude the effects of other concomitant medications due to the lack of a control group. Muscle and skin disease appeared to reduce with infliximab treatment, with improvement in CMAS and MMT8 and the modified DAS. Importantly, we observed a reduction in steroid dose after 12 months of treatment with anti-TNF, which may suggest a possible steroid-sparing effect.

Major limitations of this study include missing data (commonly encountered in multi-site cohort studies) and the lack of a control group. We recognize that it is a limitation of our study that controls were not able to be included in the analysis. We have previously used observational data to model efficacy of cyclophosphamide, another second-line treatment for JDM, using the MSM method which allowed us to compare disease activity in patients treated with the drug to those who were not treated with the drug [20]. However, this method was not feasible in this study because we included two different drugs (infliximab and adalimumab) and treatment durations were heterogeneous. Obtaining evidence for second-line treatments in this rare disease is challenging, and there is an important role for observational studies and large case series like this study. Therefore, we cannot exclude effects of concomitant medications etc.

Infliximab was switched to adalimumab in 15 patients (25%). Ten (66.7%) of the switches were due to incomplete control of disease, mainly due to lack of skin disease control. The remaining causes for switching were adverse effects and patient preference.

Calcinosis remains a significant source of morbidity for many JDM patients, yet it is poorly understood and lacks uniform treatment approaches compared to other aspects of JDM. A recent study published by the CARRA group [21] emphasizes the inconsistency in the published literature regarding therapeutic effectiveness. The authors suggest that this can be explained by the relative inexperience of physicians and the multitude of different treatments and treatment scenarios [21]. In our study, there was a reduction in the number and/or size of calcinotic lesions in 54% of the patients that were on infliximab/adalimumab and calcinosis completely resolved in 29% of them, although we cannot attribute these effects to anti-TNF treatment alone. It was also noted that the reduction in number and size of calcinosis needed nearly 2 years on anti-TNF treatment. Although we had a patient that showed evident reduction on calcinosis lesions only after 3 months on anti-TNF treatment, the majority needed a much longer time and in one case it was necessary to continue 10 years of anti-TNF treatment until the calcinosis completely resolved. This highlights that an immediate improvement on the calcinosis should not be expected, and the anti-TNF treatment should not be stopped if an immediate improvement is not seen.

Regarding safety, the majority of the adverse events were mild to moderate and mostly due to infections causes. However, we had 12 serious adverse events, mostly (*n* = 9) related with allergic reactions to infliximab. Administration of infliximab is associated with a well-recognized risk of infusion-related adverse events. The exact aetiology and pathogenesis of those infusion reactions are often unclear, and findings regarding their allergic/immune nature are inconsistent [22]. Overall the anti-TNF drugs seemed to be well-tolerated in our population with an incidence of 5.7 serious adverse reactions by 100 patient-years, which is consistent with the literature on adult patients [23]. Importantly, the majority of patients had no adverse effects and tolerated the infliximab/adalimumab well, and in the context of patients needing long-term treatment, this could be of benefit.

This study is one of the largest to describe the use of infliximab and adalimumab in a large national cohort of JDM patients. Further clinical studies are required to assess the efficacy of anti-TNF treatment in JDM, controlling for the effects of other DMARDS, and to ascertain the optimum timing for treatment initiation and cessation. Stratified analysis by autoantibody subgroups could be addressed in future studies with greater numbers.

## Conclusions

Reductions in muscle and skin disease, including calcinosis, were seen following treatment with infliximab and adalimumab. Infliximab and adalimumab were well-tolerated and the majority of patients had no adverse effects.

## Supplementary information

**Additional file 1: Supplementary Figure S1.** Flow diagram to outline which patients were included or excluded in the study and which analyses they were included in. Supplementary Results.

## Data Availability

Not applicable

## Abbreviations

CMAS: Childhood Myositis Assessment Scale
JDRG: Juvenile Dermatomyositis Research Group
MMT8: Manual Muscle Testing
PGA: Physician’s global assessment
DAS: Disease activity score
JDM: Juvenile dermatomyositis
IIM: Idiopathic inflammatory myopathy
IVIg: Intravenous immunoglobulin
JDCBS: Juvenile Dermatomyositis Cohort and Biomarker Study
DMARD: Disease-modifying anti-rheumatic drug
PRINTO: Paediatric Rheumatology International Trials Organisation
IMACS: International Myositis Assessment and Clinical Studies Group
ANOVA: Analysis of variance
HACA: Human anti-chimeric antibody
