# Supplementary material for: Retrospective analysis of infliximab and adalimumab treatment in a large cohort of juvenile dermatomyositis patients
Source: Arthritis Res Ther. 2020 Apr 15;22:79. doi: 10.1186/s13075-020-02164-5 (PMC7161150; doi:10.1186/s13075-020-02164-5)
Supplement: Supplementary file 1 — Additional file 1: Supplementary Figure S1. Flow diagram to outline which patients were included or excluded in the study and which analyses they were included in. Supplementary Results. [file 13075_2020_2164_MOESM1_ESM.docx]

**Supplementary Methods**


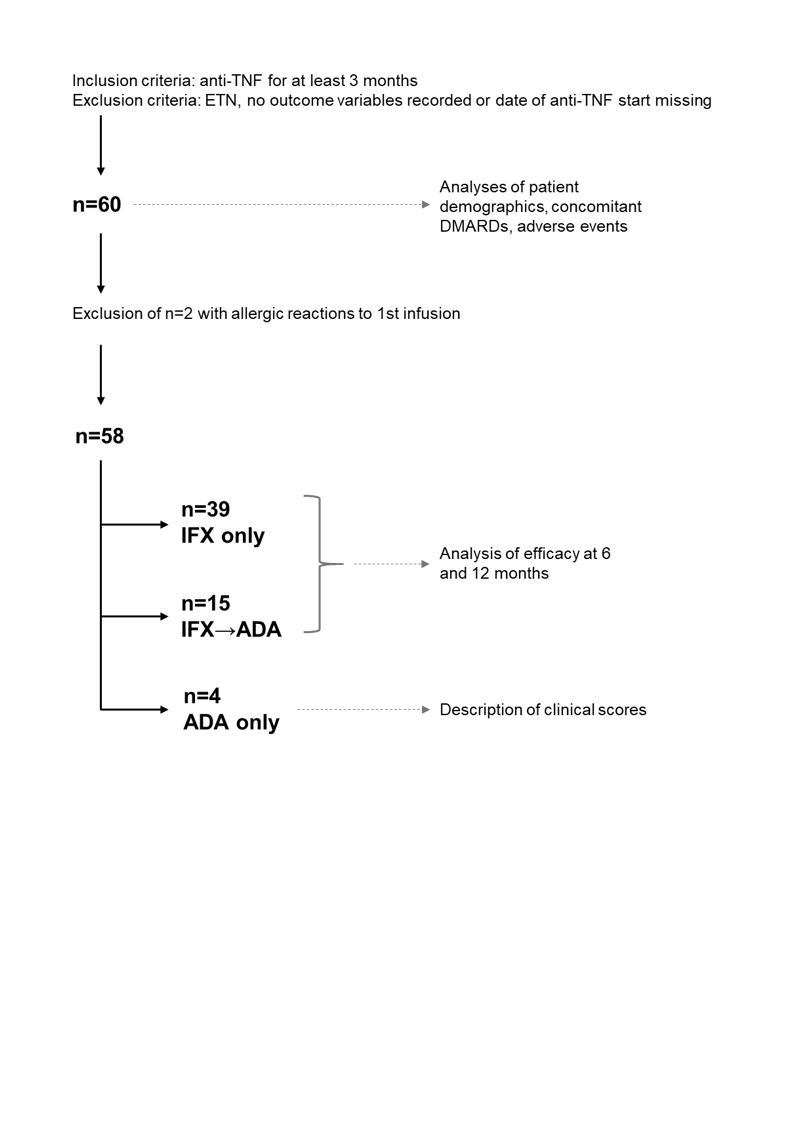


**Supplementary Figure S1.** Flow diagram to outline which patients were included or excluded in the study and which analyses they were included in.

**Supplementary Results**

Eight patients were identified who were treated with Etanercept and had complete data on the date of treatment start. Etanercept was used in patients with recalcitrant disease and non-responsive calcinosis. Modest improvements were observed in global disease, skin disease and muscle disease at approximately 12 months after Etanercept start. PGA was 1.2 [0.4-2.3] at Etanercept start and reduced to 0.6 [0.1-1.3]. Modified DAS reduced from 3 [2-4] to 1 [0.8-1.5], and CMAS reduced from 46 [45-48] to 51 [48-53].

Of these 8 patients, 3 subsequently received Infliximab. Complete data were available for skin disease activity only, with modified DAS scores of 5 [3-5] at Infliximab start and 1 [0.5-2] at 12 months after Infliximab start. One of these patients subsequently switched to Adalimumab.
